# Percutaneous treatment of valvular heart disease: Die another day

**DOI:** 10.1007/s12471-017-1044-6

**Published:** 2017-09-27

**Authors:** R. J. de Winter

**Affiliations:** Academic Medical Center, University of Amsterdam, Amsterdam, The Netherlands

A mere ten years ago, elderly patients with symptomatic severe aortic stenosis or mitral regurgitation who were considered too high risk for surgical intervention faced a sombre future. In general, exercise capacity and overall quality of life were severely impaired and treatment options were limited to symptom relief. Life expectancy was reduced, in particular when aortic stenosis occurred in combination with coronary artery disease or impaired left ventricular function. Less dramatic, but equally debilitating, was the presence of severe mitral regurgitation resulting in heart failure and dyspnoea on exertion. This last decade, a technological revolution has taken place, and now patients who are poor candidates for surgery can be treated for severe aortic stenosis with percutaneous techniques such as transcatheter aortic valve implantation (TAVI) or Mitraclip for mitral regurgitation. Predominantly elderly patients now have an option to improve their quality of life and live longer. The techniques are gradually being introduced for patients who are not considered to be so high risk for surgery, expanding the indication and widening the number of patients who qualify for percutaneous valve procedures. Cardiothoracic surgeons and interventional cardiologists are teaming up to lead this field to a better, tailored approach to valvular heart disease.

In this issue of the journal, Mart Groot et al. describe an interesting retrospective study of preoperatively screened patients undergoing noncardiac surgery who were referred for cardiology consultation between November 2011 and January 2014 [1]. Of 24,174 patients, 273 (1%) were referred to a cardiologist. The most common reason for referral (95 patients, 35%) was a request for evaluation of valve abnormalities because a murmur was present. In most patients an ECG was taken and in 192 patients (72%) a transthoracic echocardiogram was performed. In 167 patients (61%) no change in therapy was initiated, in 19% beta-blocker therapy was started and only 2% of the consultations led to an invasive procedure. The average delay of surgery after cardiology consultation was two weeks. The authors suggest that there is room for more efficient consultation, such as training anaesthesiologists to better assess cardiac murmurs by performing transthoracic echocardiography. They conclude that in most cases, preoperative cardiac consultation does not change therapy but delays surgery.

In another paper, Guiseppe D’Ancona et al. describe 14 patients who were treated in a tertiary referral centre in Rostock, Germany for combined severe aortic stenosis and severe mitral regurgitation [2]. They show that a staged approach with a TAVI procedure to alleviate the aortic stenosis, followed by treatment of the mitral regurgitation with Mitraclip an average of 101 days after the TAVI procedure, seems to be a successful approach. Although initially successful, the recurrence of symptoms leading to repeat hospitalisation is high and usually secondary to further cardiac decompensation and progressive worsening of mitral regurgitation. The authors hypothesise that technical improvement of percutaneous techniques and earlier referral for treatment could contribute to the improvement of the mid- and long-term outcomes in these complex patients.

So what connects these two papers? Well, to me, they address the two ends of the spectrum of valvular heart disease: early detection of valve disease when patients are screened for noncardiac surgery and percutaneous treatment of critically ill patients with combined severe aortic stenosis and mitral regurgitation. Although Groot et al. suggest that in many patients referred for cardiac consultation, therapy is not altered and, therefore, the consultation may be unnecessary, I would argue that this provides an excellent opportunity to detect developing valve disease at an early stage, in particular in elderly patients. Besides reducing the perioperative risk in such patients, a comprehensive assessment of their cardiac status including high-quality transthoracic echocardiography could be an opportunity to follow these patients. Elderly patients often attribute their diminishing exercise capacity and shortness of breath to advancing age. With valve disease progressing unnoticed, such patients frequently seek consultation when left ventricular function is already deteriorating and secondary mitral regurgitation is developing. Thus, I would not recommend teaching anaesthesiologists to perform (simple, screening) transthoracic echocardiography but to refer such patients. Current guidelines recommend echocardiographic examination in patients with suspected valvular heart disease in the perioperative work-up [3]. The cardiologist can not only assess the severity of valve dysfunction, but also all the other aspects of cardiovascular risk and prognosis beyond the scheduled noncardiac surgery.

Percutaneous treatment of valvular heart disease is at the brink of a whole new era. Not only are TAVI procedures evolving into highly efficient, low-risk interventions, routinely performed without general anaesthesia, with better valves, smaller sheath sizes, less need for pacemaker implantation and lower residual aortic regurgitation rates. Minimally invasive transthoracic approaches are available for patients with poor peripheral access and hybrid procedures, combining surgical and interventional approaches, are increasingly applied [4]. Valve durability, an issue with biological implants, will improve with better preservation techniques. Importantly, a whole plethora of new technologies is being actively developed for percutaneous mitral valve implantation, percutaneous mitral annulus plication, percutaneous tricuspid annulus plication, tricuspid valve clipping, and percutaneous tricuspid valve implantation, many of which are now undergoing clinical evaluation in patients. The future holds great promise for the treatment of patients with valvular heart disease and the next decade will be even more exciting.
